# A Network Convergence Zone in the Hippocampus

**DOI:** 10.1371/journal.pcbi.1003982

**Published:** 2014-12-04

**Authors:** Bratislav Mišić, Joaquín Goñi, Richard F. Betzel, Olaf Sporns, Anthony R. McIntosh

**Affiliations:** 1 Rotman Research Institute, Baycrest Centre, Toronto, Canada; 2 Department of Psychology, University of Toronto, Toronto, Canada; 3 Department of Psychological and Brain Sciences, Indiana University, Bloomington, Indiana, United States of America; Brain and Spine Institute (ICM), France

## Abstract

The hippocampal formation is a key structure for memory function in the brain. The functional anatomy of the brain suggests that the hippocampus may be a convergence zone, as it receives polysensory input from distributed association areas throughout the neocortex. However, recent quantitative graph-theoretic analyses of the static large-scale connectome have failed to demonstrate the centrality of the hippocampus; in the context of the whole brain, the hippocampus is not among the most connected or reachable nodes. Here we show that when communication dynamics are taken into account, the hippocampus is a key hub in the connectome. Using a novel computational model, we demonstrate that large-scale brain network topology is organized to funnel and concentrate information flow in the hippocampus, supporting the long-standing hypothesis that this region acts as a critical convergence zone. Our results indicate that the functional capacity of the hippocampus is shaped by its embedding in the large-scale connectome.

## Author Summary

The hippocampus is a key structure in the brain, particularly for memory function. The functional anatomy of brain networks suggests that the hippocampus should be a hub in the large-scale connectome, yet so far quantitative network analyses have failed to find any evidence of such a role. Here we show that when communication dynamics are taken into account, the hippocampus is a critical hub in the connectome. Using an anatomically realistic structural network and a novel computational model, we demonstrate that the topology of the brain is organized in a way that allows information to be funneled towards the hippocampus. Despite the fact that the hippocampus is neither among the most connected or most central nodes in the network, it receives a disproportionately high level of information flow and acts a convergence zone for the network. This convergence effect demonstrates that the architecture of the cerebral cortex facilitates integration and binding of information in the hippocampus.

## Introduction

The hippocampal formation is among the most studied areas of the brain. Along with adjacent cortical structures, such as the entorhinal, perirhinal and parahippocampal cortices, the hippocampus is thought to facilitate memory function, particularly the initial encoding of memories [1]–[4]. Lesion studies in various model organisms and in humans, as well as functional neuroimaging studies, have consistently found that the hippocampal formation appears specialized for forming conjunctions between arbitrarily different external events.

The functional anatomy of the brain supports the notion that the hippocampus may be a central structure that serves to bind together information from distributed sites in neocortex to represent a memory. Sensory information converges upon the hippocampus via multisynaptic projections, such that all fields of the hippocampus receive polysensory input from association areas of the neocortex, via perirhinal and parahippocampal cortices. In particular, the final field of the hippocampus, CA1, receives multimodal input. A prominent idea is that the hippocampus is a “convergence zone” in the brain, whereby successive levels of convergence culminate in maximally integrative regions, such as medial temporal lobe cortices [5], [6]. Altogether, the literature suggests that the hippocampus should occupy an important position in the whole-brain network.

However, despite the prominent role of the hippocampus in memory function, quantitative analyses of anatomical and functional whole-brain networks have largely failed to demonstrate the topological centrality of the hippocampus. Recent graph-theoretic studies of the connectome, both in humans [7]–[9] and in the macaque [10]–[13] have consistently found that the hippocampus is unlikely to be a hub in the context of the whole brain, as it is not among the most highly connected areas, nor does it appear to occupy a position along many of the shortest paths in the network. Likewise, the hippocampus is not highly central in large-scale functional networks [14]–[16] and even large-scale computational models have found no evidence to suggest that the hippocampus is central in the context of the whole-brain connectome [11], [17].

However, these studies have largely focused on the anatomical connectivity of brain networks, rather than on how that connectivity supports communication. In a recent report, we used a novel computational model to show that the anatomical connectivity shapes and constrains the communication dynamics of the network [18]. Interestingly, our results also suggested that the final field of the hippocampus, CA1, may be particularly important for communication, as it was the only area outside of the a set of highly connected hub nodes that was significantly over-congested relative to a null model. In the present study we show that when the communication capacity of the whole-brain network is taken into account, the hippocampus becomes a critical area, above and beyond its ostensibly average topological attributes.

## Results

**Figure 1:**
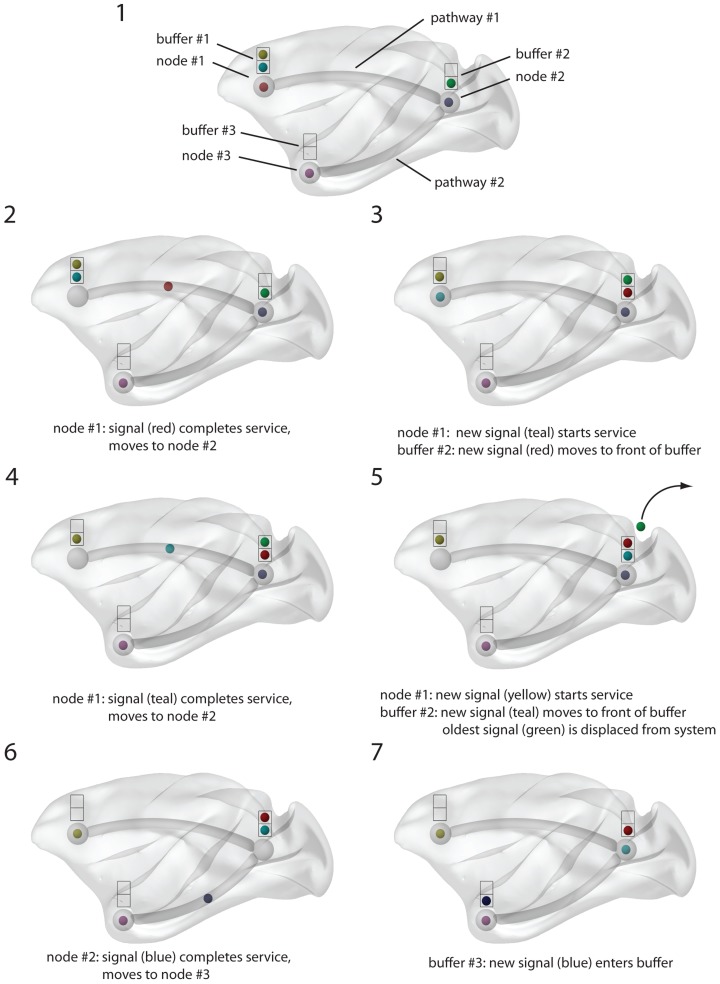
Discrete-event simulation. Schematic showing the propagation of two signal units in a simple 3-node, 2-pathway network.

To investigate the role of the hippocampus in large-scale network communication, we modeled a macaque brain anatomical network as a communication system. The structural network was derived from the online Collation of Connectivity data on the Macaque brain (CoCoMac) [19], [20], while information flow was modeled as a discrete-event queueing network [18], [21](Fig. 1). Signal units were continually generated at randomly-selected grey matter nodes in the network and assigned randomly-selected destination nodes. They then diffused through the network via white matter projections. Grey matter nodes were modeled as servers with a finite buffer capacity, such that if a signal unit arrived at an occupied node, a queue was formed. Upon reaching its destination node, the signal unit was removed from the network.

The primary goal of this approach was to generate explicit communication metrics for individual nodes in the network and to investigate the extent to which those metrics are the product of how individual nodes are embedded in the network. Information flow at individual nodes was summarized by three distinct but complementary metrics: the total number of signal units that arrive to a node (**arrivals**), the mean number of signal units at a node (**node contents**) and the proportion of time a node is occupied (**utilization**).

**Figure 2:**
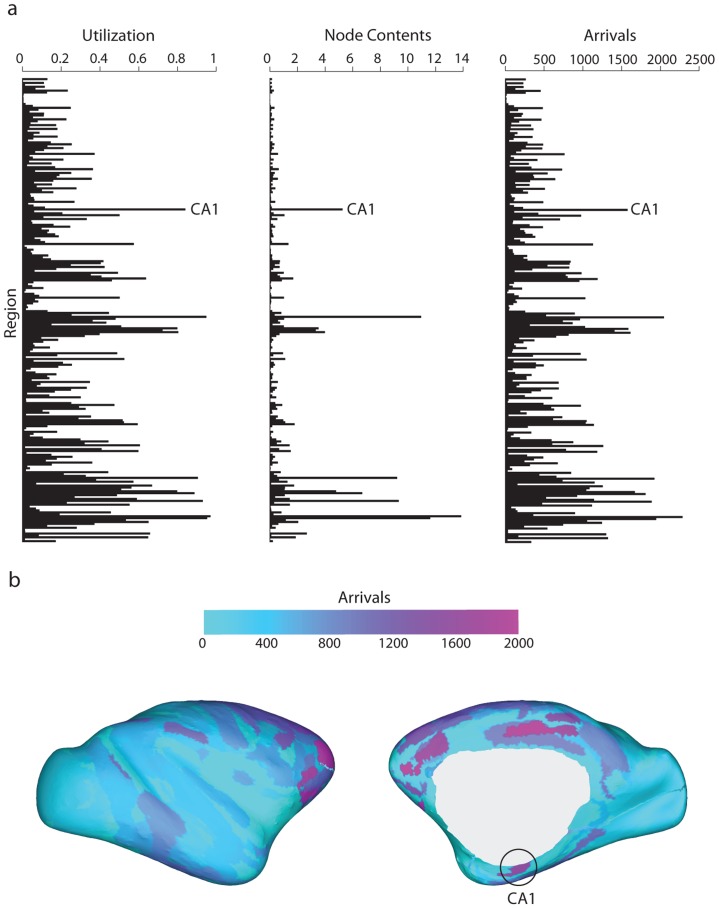
Node metrics. (a) Three local metrics of communication efficiency (utilization, node contents and arrivals) and information flow are shown for all 242 nodes of the network. averaged over 500 simulations (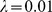, 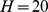, 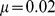). (b) Inflated surface renderings showing the anatomical distribution for the arrivals statistic, for the lateral and medial surfaces.

The first step of the analysis focuses on various communication metrics for node CA1 relative to the rest of the network. Notably, CA1 experiences a high throughput of signal traffic (Fig. 2). Fig. 2A shows the complete information flow profile for the network, with mean utilization, node contents and total arrivals at each node, while Fig. 2B shows the spatial distribution of total arrivals. For all three metrics, CA1 is ranked #7 out of 242 nodes, placing it in the top 3%. Note that there are several other brain regions that experience high traffic, including areas 13a (anterior insular cortex), 32 (anterior cingulate cortex), 23c (ventral posterior cingulate cortex) and 31 (dorsal posterior cingulate cortex). These high-degree areas are part of the putative rich club of hub nodes and their role in network communication is explored in another report [18].

**Figure 3:**
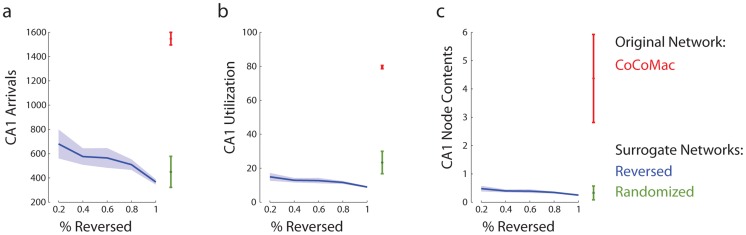
Role of network topology and directionality. The mean and standard deviation of CA1 node metrics: (a) arrivals, (b) utilization and (c) node contents. Data represent 100 simulations on the original macaque brain network (red), a single simulation for 100 randomized surrogate networks (green) and a single simulation for 100 surrogate networks with randomly reversed directions (blue).

To determine whether the high ranking of CA1 is due to its degree or due to its embedding in the global topology, the data from the macaque network were compared against a “null” model, comprised of randomized surrogate networks. In these surrogate networks the degree sequence is preserved but the global topology is destroyed by randomization. Critically, when the network is randomized, information flow through CA1 is greatly reduced (Fig. 3). The extent to which the role of CA1 differs when embedded in a randomized network versus the macaque network can be quantified and statistically assessed by expressing the mean of the macaque network distribution (red) as a 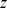-score relative to the randomized null distribution (blue). In the present case, the scores for the node contents, arrivals and utilization were 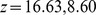 and 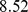 respectively, corresponding to 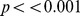.

To determine whether the directionality of cortical projections contributes to the high signal traffic at CA1, the directions of all projections in the network were reversed. We created a range directional null distributions, by reversing 20, 40, 60, 80 and 100% of all unidirectional projections in the CoCoMac adjacency matrix (Fig. 3). In other words, projections 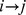 were altered such that 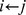. In the new network, the congestion at CA1 disappears, suggesting that the directionality of all projections in the network serves to funnel signal traffic to CA1.

**Figure 4:**
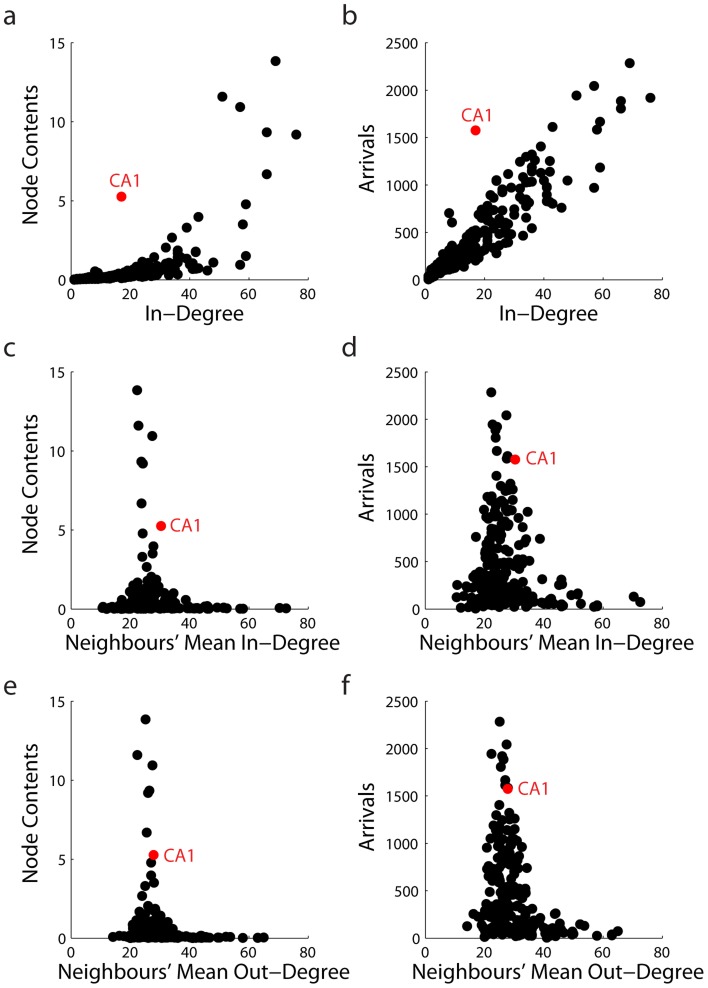
CA1 as a communication outlier. Communication metrics (node contents and arrivals) are compared to connectivity metrics, including in-degree (a,b), neighbours' mean in-degree (c,d) and neighbours' mean out-degree (e,f). In panels c-f, “neighbours” refers to nodes that project to CA1.

Thus, comparisons with null models confirm that the convergence of signal traffic at CA1 is not due to its degree, but some other higher-level feature of macaque connectivity. Specifically, the convergence of signal traffic appears to be related to both the topology and the directionality of the network. Figs. 4A and B confirm this, showing that CA1 behaves in a unique way. While greater in-degree (i.e. number of afferent projections) is associated with greater signal traffic, CA1 is a clear outlier. Namely, CA1 attracts signal traffic to an extent that is above and beyond what would be expected on the basis of its in-degree alone.

**Figure 5:**
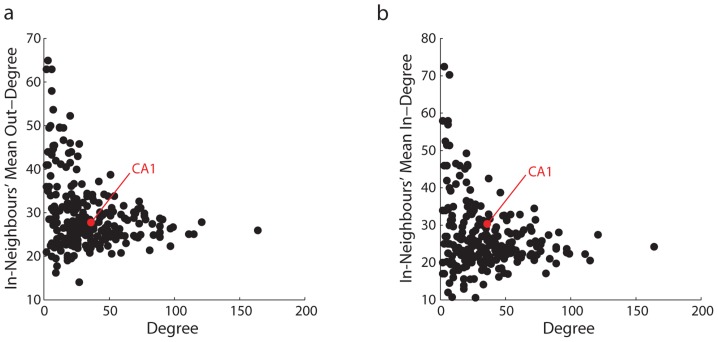
Assortativity. The degree of each node is compared to its neighbours' mean out-degree (a) and in-degree (b).

It is possible that the communication profile of CA1 is not determined by its own degree properties, but by the connectivity in its local neighbourhood. Given the diffusive dynamics of the present model, it may be that signal traffic converges to CA1 because of the nodes that project to it (i.e. its in-neighbours). One possibility is that the in-neighbours of CA1 collectively have a higher than average in-degree, and that CA1 is statistically more likely to experience higher levels of signal traffic. A second possibility is that the in-neighbours of CA1 collectively have a lower than average out-degree, thus funneling signal traffic to CA1. The plots in Figs. 4 C-F explore these possibilities and suggest that neither is likely, because neither the mean in-degree nor out-degree of the in-neighbours of CA1 is able to explain the high levels of signal traffic at that node. Furthermore, the assortativity plots in Fig. 5A and B suggest that the in-neighbours of CA1 have neither higher than expected in-degree nor lower than expected out-degree.

**Figure 6:**
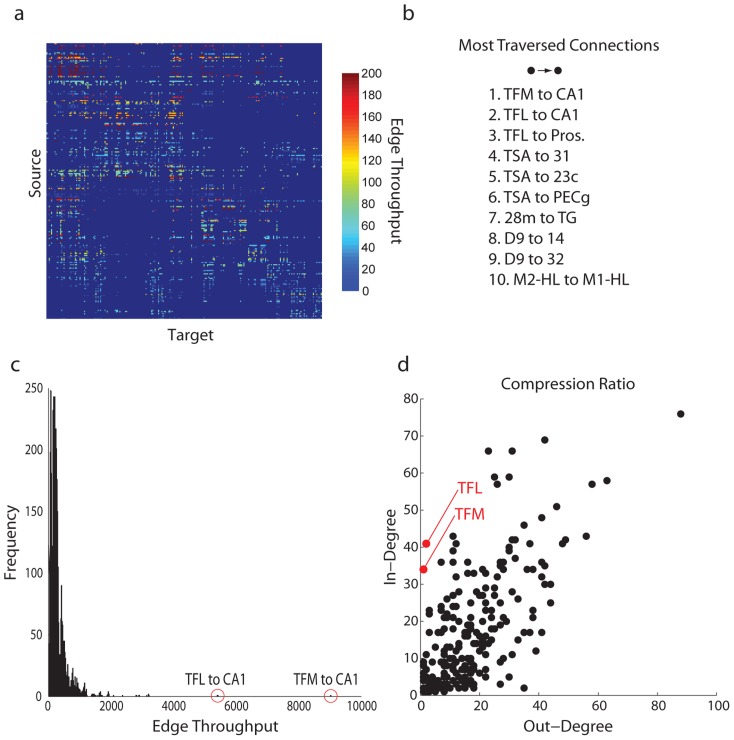
Degree imbalances. (a) The total number of signal units that traversed a particular connection. (b) The ten most traversed connections. (c) A histogram of all connections in (a), showing the distribution of signal traffic on all connections. (d) The relationship between in-degree and out-degree for all nodes in the network.

However, it may be possible that the mean connectivity profile of the in-neighbours of CA1 obscures the contribution of individual projections. For instance, it may be the case that there is a small number of projections terminating in CA1 that carry high levels of signal traffic. The total throughput along each directed connection in the macaque network is shown in Fig. 6A, while Fig. 6B shows the top 10 most traversed connections (i.e. highest-valued elements in the matrix). Note that the two most traversed connections in the network are from TFM to CA1 and from TFL to CA1. TFM and TFL represent the medial and lateral portions of parahippocampal area TF, and are known to project directly to CA1 [22]–[24]. The histogram in Fig. 6C helps to get a sense of the contribution of these two projections. The two projections are not only the most traversed; they are far removed from the distribution, representing extreme outliers.

**Figure 7:**
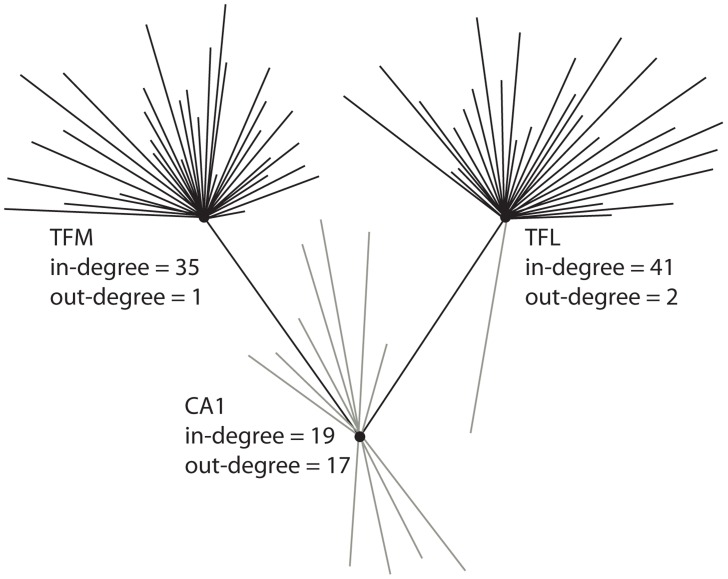
Neighbourhood of CA1. Nodes TFM and TFL have large in-degrees and low out-degrees, causing traffic to be funneled towards CA1. The nodes are spatially positioned in a way that coincides with the directionality of edges, i.e. information is projected from top to bottom.

The most plausible reason why these two projections carry so much signal traffic is because of a severe degree imbalance. Namely, both TFM and TFL have relatively large in-degrees (34 and 41, respectively), and relatively low out-degrees (1 and 2, respectively) (Fig. 6D). Thus, TFM and TFL absorb high levels of signal traffic but - as they project only to CA1 and one other node (prosubiculum) - create a funneling effect, resulting in a convergence of signal units at CA1. This degree imbalance is illustrated in Fig. 7, which shows the connectivity between TFM/TFL and CA1. Both TFM and TFL have very large in-degrees but very low out-degrees, causing traffic to be funneled towards CA1.

## Discussion

The present results demonstrate that to assess the functional capacity of the brain, it is important to consider communication dynamics, above and beyond static connectivity. Importantly, the CA1 field of the hippocampus appears to be a critical node, embedded in the connectome in a way that allows signal traffic to converge from multiple distributed areas.

These results are consistent with the notion that the hippocampal formation is a convergence zone for multiple information streams, giving rise to polysensory, multimodal representations [25], [26]. Our data suggest an integrative role, wherein information from diverse afferents is pooled and presumably integrated to engender a coherent, multimodal representation. Although the functional anatomy is highly suggestive of this role, to our knowledge the present network communication study is the first to provide quantitative topological evidence of such an organizational principle.

The information processing capacity of the hippocampus has traditionally been studied at the local level, with a focus on information flow and plasticity in the hippocampal formation and its local neighbourhood [27]. Our results suggest the hippocampus is also critical for information processing at the global level and that it is a central communication hub in the context of large-scale networks, building on considerable work regarding the role of hippocampal-cortical projections [28], [29]. Recent research suggests that information processing in hippocampal circuits is mediated by endogenous theta rhythms, with distinct phases in every field of the hippocampus [30], [31]. Information flow is then dynamically “routed” by a gamma rhythm riding on the theta troughs, centered at CA1 [32]. The results of the present study also point to CA1 as the critical field of the hippocampal formation, but more research is necessary to relate local oscillatory activity to global information flow.

Our data also indicate that the connectivity between hippocampus proper and adjacent cortex in the ventral temporal lobe is particularly important for information flow. In particular, the parahippocampal and perirhinal cortices are thought to be the primary route by which information is exchanged between the hippocampal formation and the neocortex. In the present study, the highest volume of signal traffic arrived to CA1 via direct projections from parahippocampal area TF rather than along the perforant path. This suggests that parahippocampal cortex (TF/TH) may act as a gateway for information flow to the hippocampus. The anatomical connectivity of parahippocampal cortex also supports this notion, with many neocortical afferents converging on this particular site [33].

More generally, the present results highlight the dynamic importance of unidirectional connections and of degree sequences [34]. Several recent studies have pointed to degree imbalances and their possible importance for information flow [13], [35], [36]. The present results confirm that large scale network communication may depend on these properties, and further illustrate why analyses of structural and functional connectomes should take into account the role directionality and degree sequences.

### Methodological limitations

In the present study communication dynamics are modeled in terms of diffusive signal traffic. This approach confers several advantages, including the ability to trace individual signal units, as well as no strong assumptions about the routing and transformation of information in the network. However, the model is also limited in some ways and it is important to consider to what extent the assumptions of the model limit the conclusions drawn from the data.

First, all areas and projections are assumed to have the same capacity, despite the fact that the former vary in size and the latter vary in fiber density. The model was configured in this way to avoid making even stronger assumptions about how size influences processing capacity. For example, it is unclear *a priori* whether the information processing capacity of an area should vary in a linear or nonlinear fashion with size. In addition, the current configuration of the model makes the results of the study comparable to conventional graph-theoretic analyses, which also assume no variation across nodes or across edges [37]–[39].

Second, all signal units are assumed to carry the same amount of information and they flow through the network unchanged. This is unlikely to be the case in real brain networks, where information is fundamentally transformed at each node [40], [41]. The model was configured in this particular way to reduce the complexity of the problem and to focus on the main experimental question: how does the topology of the network influence information flow to the hippocampus? To trace the trajectory of individual signal units it is necessary that they remain unchanged. Thus, discrete signal units represent the ability of brain areas to influence each other via anatomical projections.

Third, the present model is unable to incorporate physiologically realistic oscillations in different frequency bands. We anticipate that, given the importance of theta and gamma rhythms in the hippocampus [30]–[32], this will be an important future step. Likewise, the input to the model may be further refined in the future, to more accurately reflect 1/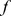 sensory inputs, as well as the contribution of thalamocortical projections.

In addition, it is important to note that our findings are only as accurate as the anatomical connectivity data on which they are based. The CoCoMac-derived anatomical connectivity matrix used in the present study is collated from a database of tract tracing studies [19], [20] and may be limited by the fact that these studies used different tracing methods, nomenclature and planes of section. In a set of recent reports, Markov and colleagues described a large-scale profile for 29 areas of the macaque brain, derived using a systematic and consistent retrograde tracing procedure [35], [36]. When this connectivity matrix is completed and includes the different fields of the hippocampus as well as parahippocampal areas, it will be possible to verify and validate the present findings which are based on the coarser CoCoMac network.

### Conclusion

The present study demonstrates that the hippocampus, particularly subfield CA1, is an important communication hub not just at the local level, but in the large-scale connectome as well. These results showcase an important principle: the functional capacity of a given region or subnetwork cannot be fully discerned by only analyzing the static structural connectivity of the brain. It is the *communication* between regions that engenders complex phenomena such as perception, cognition and action. Communication dynamics are the link between structure and function and thus represent an important attribute of brain networks.

## Materials and Methods

The data were generated using the same anatomical connectivity, queueing network model and parameters as reported in [18].

### Anatomical and reference networks

The anatomical adjacency matrix was compiled using the online Collation of Connectivity data on the Macaque brain (CoCoMac) database [19], [20], which includes information from 413 tract tracing studies in the macaque brain. The database was originally queried by [12] and subsequently refined by [13]. The resulting fully connected directed adjacency matrix included 242 nodes and 4090 edges. Importantly, the connectivity matrix contained several nodes that are part of the hippocampal formation and neighbouring cortical structures, including parahippocampal areas TF/TH, perirhinal areas 35/36, entorhinal cortex, dentate gyrus, subfield CA3, subfield CA1 and subiculum.

To determine the effect of global network topology on communication, a population of degree-matched randomized surrogate networks was generated using a Markov switching algorithm that randomly swapped pairs of edges [42]. In these randomized networks, the in-degree and out-degree of each node is preserved while the global topology is altered, allowing us to differentiate effects due to topology from effects due to degree. All statistical inference was performed by comparing 100 simulations on the CoCoMac network with 100 simulations on a randomized surrogate network (serving as a “null” network), for 100 network realizations.

### Discrete-event simulation

Individual signal units were generated in the network as a Poisson process with rate 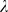, i.e. with exponentially distributed inter-arrival times. Each signal unit was generated at a randomly selected source node and assigned a randomly selected destination node. When a signal unit reached its destination node, it was removed from the network. Until it reached its destination node, the signal unit propagated to one of the neighbouring nodes. Thus, if a signal unit was at node 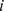, with out-degree 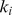, the probability of traveling to any one of the neighbouring nodes was 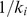. The time spent at each node (service time) was exponentially distributed with rate 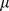.

Signal units that arrived at an occupied node were placed in a buffer and formed a queue. Signal units queued on a last-in-first-out (LIFO) basis, also known as last-come-first-served (LCFS) [43]–[45]. The buffers had limited capacity (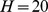), such that signal units that arrived at a full buffer caused the last signal unit in the queue to be ejected from the buffer and removed from the system.

Fig. 1 illustrates how a discrete-event simulation works. In this example, there are three nodes, each of which has a two-slot buffer, that are interconnected by two pathways. At time 2, a signal (red) completes service at node 1 and moves to node 2. The signal (red) arrives at node 2 and enters the buffer. Due to the last-come-first-served queueing discipline, the signal moves to the front of the buffer. Meanwhile, the signal (teal) in the buffer at node 1 commences service (time 3). At time 4, the signal (teal) at node 1 completes service and moves to node 2, where it moves to the front of the buffer and displaces the oldest signal (green), which is ejected from the network (time 5). At time 6, the signal (blue) at node 2 completes service and moves to node 3, where it enters the empty buffer (time 7). Meanwhile, the signal (teal) at the front of the queue at node 2 commences service.

All simulations had a duration of 2 million dimensionless time units. System state was updated at non-uniform intervals due to the presence of stochastic variables (signal inter-arrival time and service time). To allow further analysis, the time series of system states were re-sampled at uniform intervals using linear interpolation (“table lookup”). Using the ensemble average method [44], an initial transient of 40,000 time units was identified and removed from further analysis. The exponentially distributed random variables (inter-arrival time and service time) were generated by the inverse transform method, using the Mersenne Twister to generate a uniform distribution for input [46].

### Communication metrics

Node-specific communication metrics were defined as follows. For node 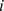, the total number of **arrivals** during a simulation run included both the exogenous Poisson arrivals, as well as arrivals from afferent projections. Node 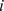 at time 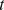 consists of two parts: a server with contents 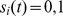 which corresponds to whether there is a signal unit currently in service, and the queue length 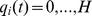, which corresponds to the number of signal units in the buffer. The **node contents** 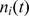 are the sum of the server and queue contents
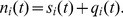
(1)


Finally, the **utilization** of node 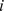 is proportion of simulation time that 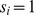.

### Mapping parameter space

Parameter space mapping is described in detail in [18], but for completeness is reviewed here. The key consideration is that this type of stochastic model has two modes of operation. System behaviour is primarily determined by the ratio of the external arrival rate (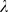) and the service rate (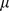) at each node. When the external arrival rate: service rate ratio is low, the total number of signal units in the network is stable and the system is in a steady state. As the external arrival rate: service rate ratio is increased, the system undergoes a phase transition to a jammed state, such that the total number of signal units in the network increases monotonically until all buffers are filled to capacity [47], [48].

The focus of the present study was on the steady state behaviour of the network, so the parameters were chosen to sustain stationary flow, prior to the phase transition, following the findings presented in [18]. The service rate (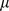) and the rate of external arrivals (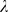) were fixed at 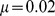 and 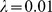. The results of the present analysis hold for a wide range of external arrival rates (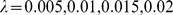), but for simplicity only this point in parameter space is shown. For this type of dynamic system, buffer capacity (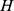) is not a critical parameter, because it cannot induce a phase transition. For the present set of simulations, buffer capacity was set to 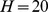.

### Physiological foundation

The discrete-event stochastic model entails a number of simplifying assumptions and it is important to clarify what aspects of neural physiology are captured by different features of the model.

**External arrivals** to the network represent the assumption that information is perpetually generated in brain networks, either due to external stimulation or some endogenous process. **Poisson arrivals** were informed by research in psychophysics. In the psychophysics and signal detection literature, statistical fluctuations of the sensory environment (e.g. photons impinging on the retina, energy fluctations in auditory stimuli) display Poisson statistics and/or are well-fitted by Poisson process models [49]–[52].

**Discrete signal units** represent the ability of brain regions to influence one another and may be thought of as perturbations that spread through the network. Physiologically, these perturbations may correspond to spike trains or coordinated volleys of spike trains. In addition, for discrete signal units (as opposed to continuous information flow), it is possible to monitor the trajectory of information flow in the network and to investigate how the anatomical connectivity facilitates communication. The purpose of **queues** and **finite buffers** is to model how information flow is constrained by the topology of the network. Queueing is a mechanism by which signal units interact, allowing us to model how multiple information flows emerge on the anatomical network. Finite buffers ensure that signal units can be lost, which mimics the poor fidelity of information transmission in the brain [53]. Moreover, the LIFO queueing displine ensure that the oldest signals in the buffer are the first to be lost, which models the natural time-dependent decay of biological signals.

Communication in the network is mediated by **diffusion**. Diffusive dynamics seem advantageous for modeling neural information flow because this type of model does not assume that signal units have knowledge about global network topology. Moreover, a number of recent studies have shown that diffusion may be a viable mechanism for information transfer in real brain networks [54]–[56] (but see also [40], [41]).

## Supporting Information

Dataset S1
**Supplementary materials.** Simulation results are contained in three structures, representing metrics of congestion at CA1: Arrivals, Utilization and Contents. Each structure contains three fields, which correspond to the three network types: the original CoCoMac network, as well as Randomized and Reversed surrogate networks. In the case of Randomized networks, there are 5 sets of 100 simulations, which correspond to 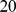, 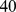, 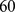, 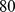 and 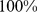 reversals of unidirectional projections. The array “CoCoMac_adjacency” contains the adjacency matrix of the CoCoMac network, while the cell “Labels” contains the labels of each of the 242 brain regions.(MAT)
